# A case of unilateral and spontaneously resolving posterior uveitis with overlapping features of Vogt–Koyanagi–Harada disease and Acute Posterior Multifocal Placoid Pigment Epitheliopathy

**DOI:** 10.1186/s40064-016-3132-2

**Published:** 2016-09-01

**Authors:** Bo Li, Ricarda J. Bentham, John R. Gonder

**Affiliations:** 1 Ivey Eye Institute, Western University, London, ON Canada; 2 University of Ottawa Eye Institute, University of Ottawa, Ottawa, ON Canada; 3 St Joseph’s Hospital, 268 Grosvenor Street, London, ON N6A 4V2 Canada

**Keywords:** APMPPE, VKH, Uveitis, OCT, IVFA, White dot syndrome, Acute Posterior Multifocal Placoid Pigment Epitheliopathy, Vogt–Koyanagi–Harada

## Abstract

**Introduction:**

VKH disease is a chronic, bilateral, granulomatous panuveitis with potential involvement of neurological, auditory and integumentary systems. On the other hand, APMPPE is believed to be an immune-driven chorioretinal vascular disease characterized by multifocal, flat, grey-white placoid lesions at the level of the RPE. We describe a case with overlapping figures of both conditions.

**Case description:**

A 19-year-old female presented with unilateral blurry vision and was found to have clinical and IVFA findings consistent with APMPPE. Her OCT study demonstrated typical VKH findings with large areas of serous neurosensory retinal detachment and intra-retinal cystoid spaces with enclosed membranous structures. She was closely followed but was not treated with high dose corticosteroid. Spontaneous and complete resolution of her symptoms and clinical, IVFA and OCT findings were achieved by day 25.

**Discussion:**

This is the first reported case of spontaneously resolving, unilateral VKH disease in the absence of high dose corticosteroid treatment with overlapping features of APMPPE.

**Conclusions:**

The imaging and clinical findings of both VKH disease and APMPPE raise the notion that VKH disease and APMPPE could be an overlapping spectrum of inflammatory processes, rather than distinct disease entities.

## Background

Vogt–Koyanagi–Harada (VKH) disease is a chronic, bilateral, granulomatous panuveitis with potential involvement of neurological, auditory and integumentary systems. Characteristic findings of VKH disease include bilateral involvement, granulomatous anterior uveitis, choroidal thickening, optic disc hyperemia and/or edema, multiple serous retinal detachments and sunset-glow fundus in the chronic phase of the disease (Fang and Yang 2008). VKH disease most commonly presents simultaneously in both eye, however, the second eye involvement may have delayed onset (Read et al. 2001). Unilateral VKH disease without second eye involvement is considered exceedingly rare. The treatment for VKH disease almost always requires high dose oral and/or intravenous corticosteroids and there are currently no reported cases of spontaneously resolving VKH disease.

Acute Posterior Multifocal Placoid Pigment Epitheliopathy (APMPPE) is believed to be an immune-driven chorioretinal vascular disease characterized by multifocal, flat, grey-white placoid lesions at the level of the retinal pigment epithelium (RPE) (Gass 1968). It is often a bilateral disease, which affects males and females equally between the ages of 20 and 40. Patients typically present with photopsia, headache, eye pain and central and/or paracentral scotoma in one eye with the involvement of the fellow eye within weeks. Approximately 50 % of patients will have prodromal flu-like symptoms prior to disease onset (Gass 1968; Kaplan et al. 2013). The diagnosis of APMPPE is typically based on clinical presentation and fluorescein angiography findings which include early hypofluorescence of the lesions followed by hyperfluorescence, with or without late staining (Crawford and Igboeli 2013). Despite the fact that most patients experience a significant deterioration in vision at the disease onset, APMPPE is a self-limiting condition with a good visual prognosis. The majority of patients will recover a visual acuity of 20/40 or better within 6 months with 20 % of patients left with residual visual dysfunction (Fiore et al. 2009).

In this report, we describe a unilateral and spontaneously resolving case of posterior uveitis with overlapping features of VKH disease and APMPPE.

## Case report

A 19-year-old female of East African descent presented with a 3-day history of paracentral blurring with constant, stabbing pain in her left eye. One week prior to the visual symptoms, the patient experienced flu-like symptoms including nasal congestion, cough, sore throat, nausea, dizziness and mildly swollen wrists. The patient was given a 4-day course of prednisone 5 mg daily by an emergency physician prior to the onset of her visual symptoms. There were no auditory, dermatological or neurological symptoms. The patient’s family history, past ocular and medical history was unremarkable.

**Fig. 1:**
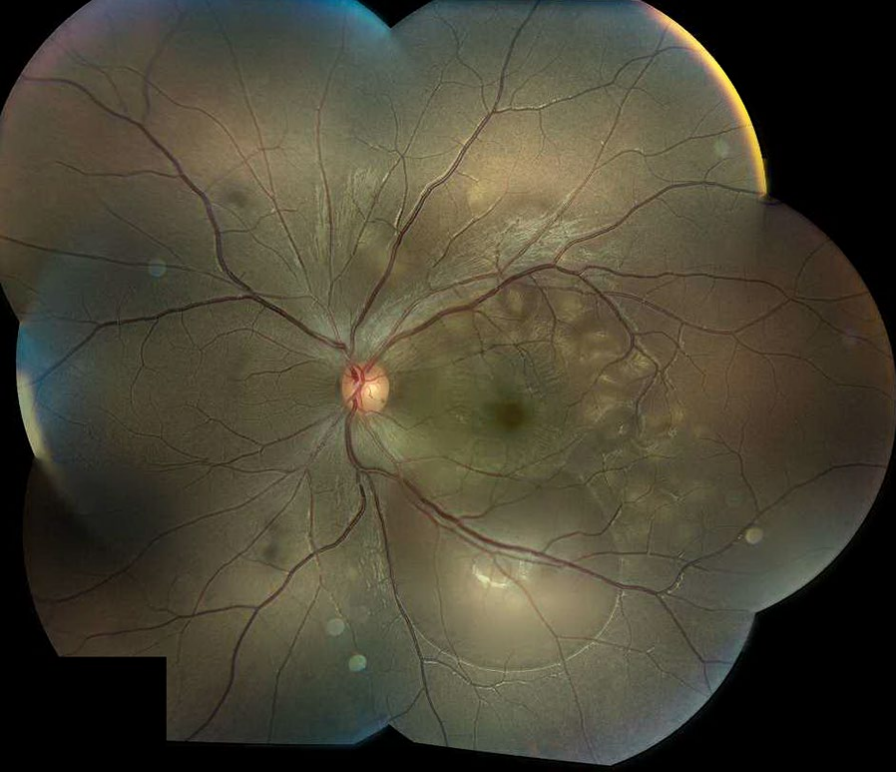
Left eye fundus photograph at the time of disease onset. Multiple, *creamy yellow*, flat, placoid lesions at the level of retinal pigment epithelium and multiple areas of retinal elevation consistent with sub-retinal fluid were visualized in the posterior pole

**Fig. 2:**
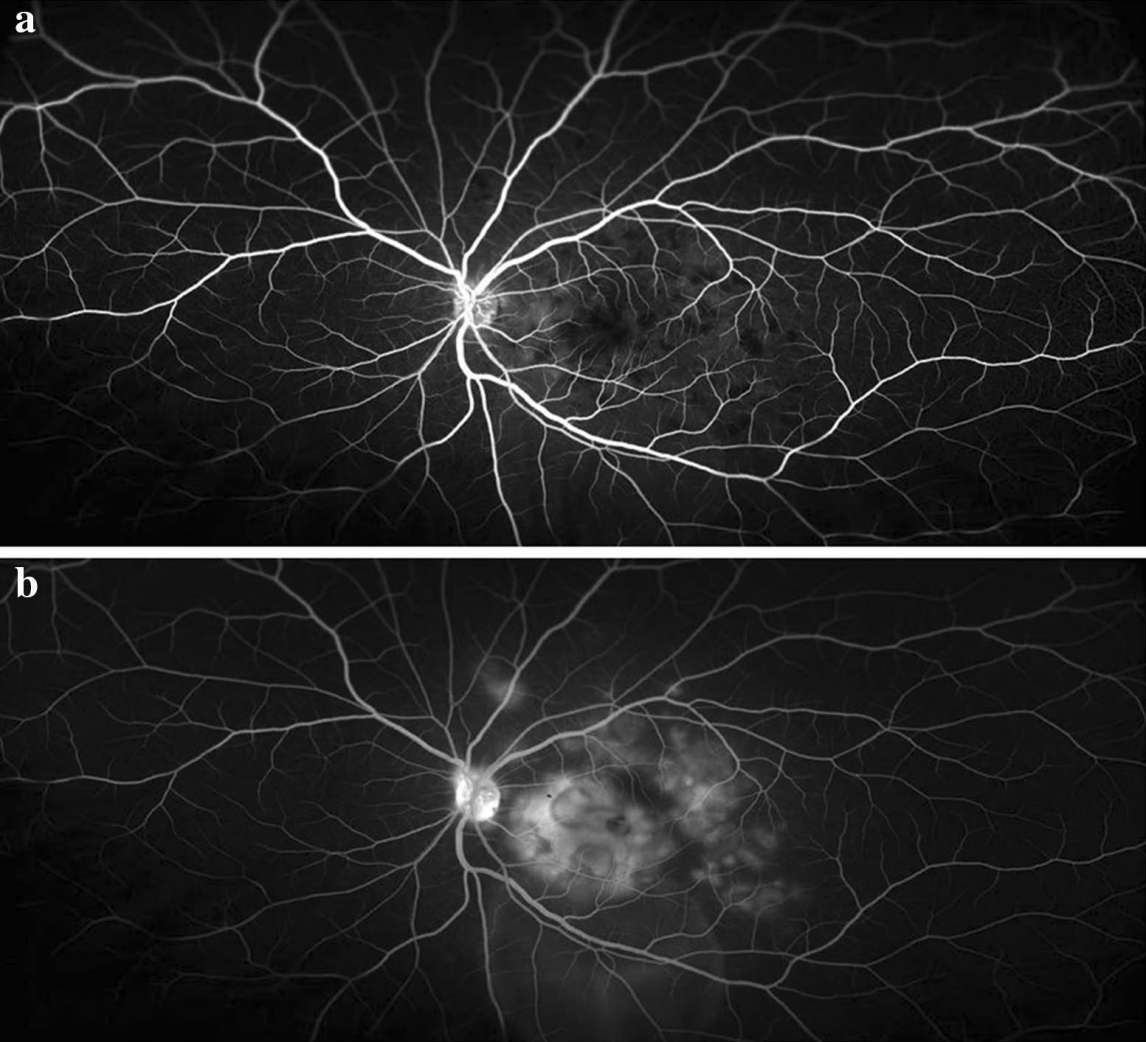
Wide-angle IVFA of the left eye at time of disease onset. **a** Arteriovenous phase showing diffuse areas of hypofluorescence more numerous than lesions seen on color fundus photograph. **b** Late phase showing late staining and leakage from areas of previous hypofluorescence and pooling over areas of serous retinal detachment

On exam, her best corrected visual acuity (BCVA) was 20/20 OD and 20/70 OS. There were no pupillary abnormalities and no evidence of a relative afferent pupillary defect. Intraocular pressure, external and anterior segment exam were unremarkable without signs of inflammation bilaterally. The dilated funduscopic exam was unremarkable for the right eye, but the left eye revealed multiple, yellow, placoid lesions and areas of retinal elevation consistent with subretinal fluid, as well mild optic disc hyperemia of the left eye (Fig. 1). A wide-angle Intravenous Fluorescein Angiography (IVFA) study demonstrated multiple small areas of early hypofluorescence limited to the posterior pole with significant late hyperfluorescence including staining, leaking, and pooling in the areas of serous retinal detachment (Fig. 2). The areas of early hypofluorescence appeared to correspond with the yellow, placoid lesions seen on fundus examination. In the early phase, there were no pinpoint areas of leakage or staining identified. Optical Coherence Tomography (OCT) showed significant disruption of ellipsoid layer and external limiting membrane, attenuation and edema of the photoreceptor segments, and large areas of serous neurosensory detachment with subretinal fluid. Unfortunately, the OCT imaging did not include Enhanced Depth Imaging function in order to assess choroidal thickness. An initial diagnosis of atypical unilateral Acute Posterior Multifocal Placoid Pigment Epitheliopathy (APMPPE) was made based on the fundus appearance and IVFA findings and the patient was followed closely without treatment. CSF analysis and HLA typing were not performed at the time given the initial diagnosis of APMPPE.

On day 3, the patient returned to clinic with worsening vision in the left eye to counting fingers and peri-orbital swelling. Anterior segment examination of the left eye showed conjunctival injection and 2+ cells in the anterior chamber and minimal cells in the vitreous. Fundus examination did not demonstrate any changes from her previous exam. The patient was started on Loteprednol 0.5 % QID to the left eye for anterior segment inflammation.

Subjective visual improvement was reported starting on day 4 with the OCT appearance of large intraretinal cystoid spaces and the appearance of membranous structures within the cystoid space (Fig. 2b). By day 12, BCVA in the left eye improved to 20/70, the ocular pain and peri-orbital swelling resolved and the topical Loteprednol was stopped. The OCT on day 12 demonstrated significant resorption of cystic spaces and subretinal fluid, as well as reduced disruption of the ellipsoid layer and photoreceptor segments (Fig. 2c). On day 25, the patient’s BCVA improved to 20/20 with some mild subjective visual distortion. The OCT showed complete resorption of the subretinal fluid, cystic spaces and reestablishment of normal retinal anatomy including the ellipsoid layer, external limiting membrane and inner and outer photoreceptor segments (Fig. 2d). At the 2 months follow up, the patient’s BCVA remained at 20/20 OU and she had an unremarkable ocular exam with the exception of subtle retinal pigmented epithelium changes. At 6 and 18 month follow-up, the patient’s BCVA remained at 20/20 OU without any visual disturbances. All the investigations for inflammatory markers, rheumatologic markers, sarcoidosis, tuberculosis and syphilis were all unremarkable.

## Discussion

VKH has been long considered to be a bilateral panuveitis. The revised diagnostic criteria published in 2001 for VKH disease defined VKH disease as a bilateral ocular inflammatory disease in the absence of ocular trauma, surgery and without evidence of other ocular or systemic disease (Read et al. 2001). To date, there have been only 13 reported cases of unilateral VKH disease without involvement of the other eye (Forster et al. 1991; Agrawal and Biswas 2011; Yokoi et al. 2010; Usui et al. 2009; Neves et al. 2015). Similarly, APMPPE is also consider a bilateral condition with the manifestation of primary choriocapillaris perfusion abnormalities with secondary involvement of the RPE, photoreceptor, and outer retinal layers (Gass 1968; Kaplan et al. 2013; Crawford and Igboeli 2013; Fiore et al. 2009; Mrejen et al. 2013; Howe et al. 1995).

In the case we reported, there is a combination of typical and atypical features for both VKH disease and APMPPE. The flu-like prodrome, placoid retinal lesions on fundus examination with early hypofluorescence and late hypofluorescence on IVFA are in keeping with an APMPPE diagnosis. The absence of the typical “starry sky” appearance seen in VKH disease, the spontaneous resolution of functional and structural abnormalities and no disease recurrence are all also typically seen with APMPPE (Steiner and Goldstein 2012). The areas of late phase staining and leakage corresponding with areas of placoid lesions are likely the areas of choriocapillaris ischemia with subsequent serous leakage into the subretinal space, as previously described in both APMPPE and VKH disease (Gaudric et al. 1982, 1987; Fardeau et al. 2007).

**Fig. 3:**
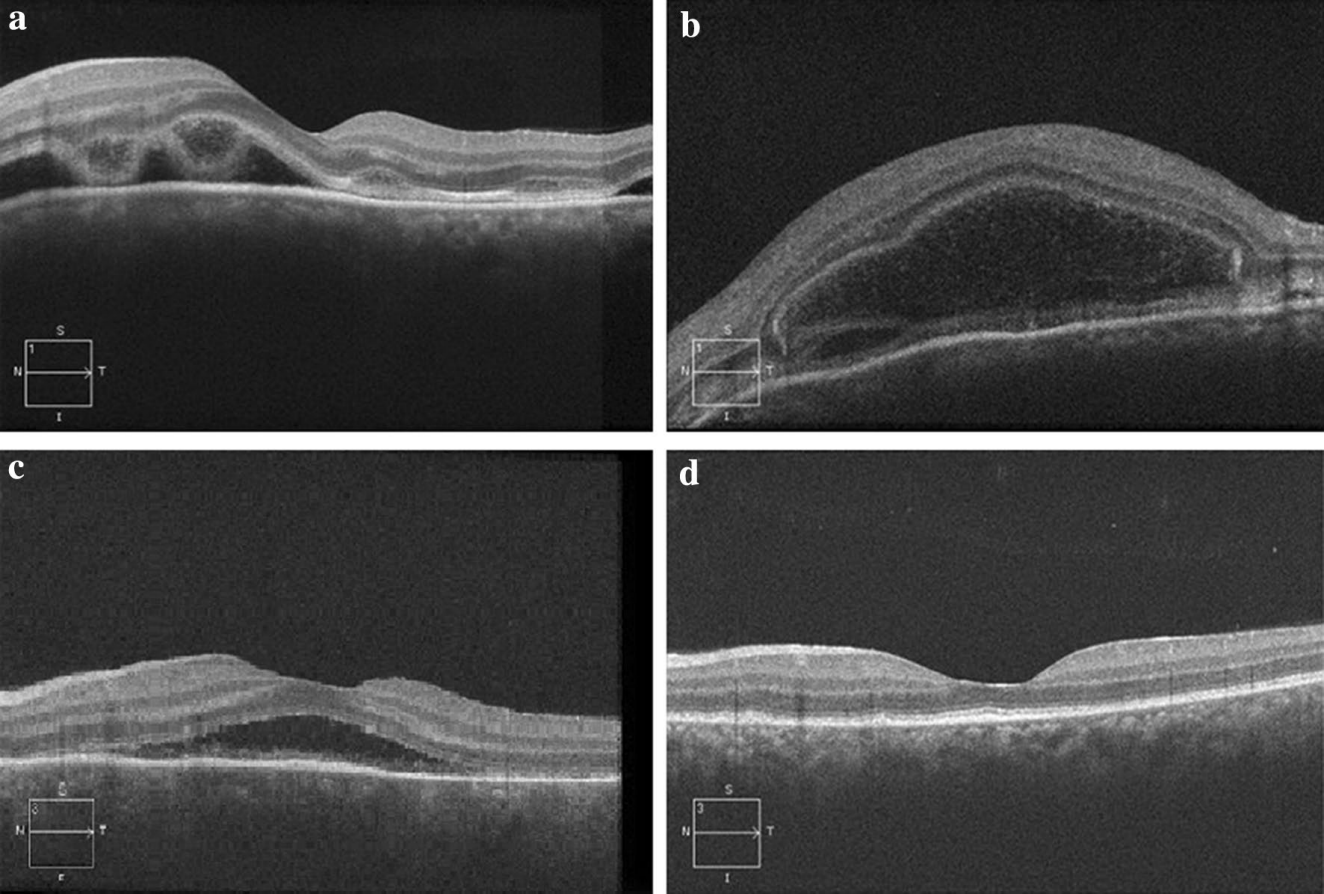
OCT images during acute, resolving and resolved phases. **a** Day 0 of disease onset, areas of serous neurosensory detachment with subretinal fluid, ellipsoid layer and external limiting membrane disruption, attenuation and edema of photoreceptor segments were seen. **b** Day 4, large intraretinal cystoid spaces and membranous structures within the cystoid space were seen. **c** Day 12, significant resorption of subretinal fluid and cystoid spaces, as well as granular appearance of the membranous structure within the cystoid spaces were observed. There was also resolving edema and reduced disruption of the ellipsoid layer and photoreceptor segments. **d** Day 25, complete resorption of cystoid spaces and re-establishment of retinal layers

 The large areas of serous retinal fluid and the OCT findings are typical for VKH disease. In the acute phase, large areas of multifocal serous retinal detachment are observed (Fig. 3a). In the recovering phase, there is coalescence of serous retinal detachments and the appearance of large cystoid spaces and a membranous structure of uniform thickness at the floor of the cystoid spaces (Fig. 3b, c). The membranous structure and its ground glass appearance are highly suggestive of VKH disease (Ishihara et al. 2009). As the recovery process continues, the membranous structure takes on a more granular appearance (Fig. 3c). Those OCT findings have been well documented as typical VKH disease progression. It is hypothesized that the membranous structure is a portion of the outer segment layer of the photoreceptors that is attached with inflammatory products such as fibrin takes on a more granular appearance as the inflammatory process lessens during recovery phase (Ishihara et al. 2009). Other typical OCT changes of the RPE and choroid layers for VKH disease include undulations of the RPE surface with the undulation troughs corresponding to areas of choroidal striations and areas of hypofluorecence on IVFA (Gupta et al. 2009). Although there were mild undulations of the RPE layer observed in the acute phrase of our case, there were no choroidal striations seen. Similarly in APMPPE, reflective changes above the RPE and on the plane of the RPE suggesting inflammatory cells with edema in the outer retinal layers and the loss of inner/outer segment (IS/OS) junction, attenuation of photoreceptor layer and attenuation of the external limiting membrane, as well as nonspecific changes in the choriocapillaris have been reported in APMPPE (Kaplan et al. 2013; Cheung et al. 2010).

What is unique in our case is the fast resolution of disease without the use high dose corticosteroid. Complete functional and structural recovery was achieved within 25 days, which is more in keeping with a diagnosis of APMPPE. Furthermore, both APMPPE and VKH syndrome are considered bilateral conditions with involvement of the fellow eye within days to weeks. The unilaterality of our case is another feature that’s not commonly seen with either disease entity.

## Conclusions

We are reporting a unique case of unilateral, spontaneously resolving posterior uveitis with IVFA and OCT findings consistent with both APMPPE and VKH disease. This is the first reported case of spontaneously resolving VKH disease with complete functional and structural resolution without the use of high dose corticosteroid. There are least two previous reports of overlapping clinical and imaging findings between VKH disease and APMEPPE (Tanigawa et al. 2013; Lee et al. 2011). The imaging and clinical findings of both VKH disease and APMPPE support the notion that VKH disease and APMPPE could be an overlapping spectrum of inflammatory processes that lead to choroidal and choriocapillaries ischemia with secondary damage to the RPE and outer retinal layer structures.
